# A Sensitive Approach for the Determination of the Novel Antidepressant Vilazodone: Electrochemical Sensing Using a Boron‐Doped Diamond Electrode in Surfactant‐Containing Media

**DOI:** 10.1002/open.70298

**Published:** 2026-10-22

**Authors:** Birdem Bersu İlvan, Ipek Kucuk, Selenay Sadak, Hülya Silah, Bengi Uslu

**Affiliations:** ^1^ Faculty of Pharmacy Department of Analytical Chemistry Ankara University Ankara Türkiye; ^2^ The Graduate School of Health Sciences Ankara University Ankara Türkiye; ^3^ Faculty of Pharmacy Department of Analytical Chemistry Başkent University Etimesgut Ankara Türkiye; ^4^ Faculty of Science Department of Chemistry Bilecik Seyh Edebali University Bilecik Türkiye

**Keywords:** boron‐doped diamond electrode, electrochemical detection, surfactant, Tween 20, vilazodone

## Abstract

A novel, highly sensitive surfactant‐supported method was developed for vilazodone (VLD) determination using a boron‐doped diamond (BDD) electrode. This study represents the first VLD detection on a BDD electrode and the first application of surfactant‐supported voltammetry for this antidepressant. The electrochemical behavior of VLD was evaluated at different pH values using differential pulse voltammetry (DPV). The peak potential shifted negatively up to pH 7.0 and stabilized thereafter, indicating a proton‐involved oxidation mechanism with a pKa near 7.0. The effects of CTAB, SDS, and Tween 20 were investigated, with Tween 20 providing the greatest peak‐current enhancement due to its nonionic character, interfacial organization, and microcolloidal environment, improving mass transport and suppressing adsorption‐controlled pathways. Under optimized conditions, excellent linearity (*R* ^2^ >0.988) was obtained over 5.0 × 10^−10^–1.0 × 10^−6^ M. Recoveries of 98%–102% were achieved in pharmaceutical formulations and spiked human serum samples, demonstrating applicability to complex matrices. Density functional theory calculations showed highest occupied molecular orbital localization mainly over the nitrogen‐containing conjugated region and partly on the piperazine moiety, supporting piperazine nitrogen involvement in anodic oxidation. The proposed DPV‐BDD platform provides a rapid, sensitive, and reliable approach for VLD analysis with nonionic surfactant‐assisted signal enhancement.

## Graphical abstract

The combination of BRBS with Tween 20 enhanced the VLD oxidation signal at ~+0.67 V through surfactant‐assisted adsorption and preconcentration on the BDD electrode surface, resulting in improved sensitivity and signal‐to‐noise ratio. In this study, VLD was also found to undergo oxidation via the piperazine ring through a 2e^−^/2H^+^ process at the boron‐doped diamond electrode.

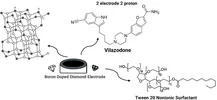

## 1 Introduction

Vilazodone (VLD), having the chemical name of 5‐(piperazin‐1‐yl)‐1‐benzofuran‐2‐carboxamide, is an antidepressant with a dual mechanism of act, combination selective serotonin reuptake prevention with partial agonism at serotonin 5‐HT1A receptors [1, 2, 3]. Thanks to its pharmacological activity and the significance of dependable drug‐level assessment, the development of sensitive and selective analytical techniques for VLD determination in pharmaceutical and biological samples remains analytically relevant.

VLD is a new antidepressant drug that combines SSRI activity and partial agonist activity of the serotonin 5‐HT 1A receptor in a single molecule. Steady‐state concentrations of VLD are reached approximately 3 days after dosing, and the maximum plasma concentration of VLD has been reported as 156.3 ng/mL. Antidepressants may cause side effects such as nausea, dizziness, insomnia, and restlessness, especially during the period until steady‐state concentrations are reached when treatment is initiated. Therefore, dose adjustment may be necessary during this period. Monitoring therapeutic drug levels in biological samples during dose adjustments allows the therapeutic range to be maintained [4]. Electrochemical methods enable the sensitive and selective detection of psychoactive drugs, such as vilazadone, even at low concentrations.

In the last 5 years, various analytical techniques, including high‐performance liquid chromatography with fluorescence and DAD detectors [5, 6], ultraperformance liquid chromatography‐electrospray ionization‐mass spectrometry (UPLC‐ESI‐MS/MS [7]), as well as fluorimetric [8] and electrochemical methods [9, 10] have been reported for VLD in dosage forms and biological matrices. Fekry et al. created a carbon paste electrode enhanced with cellulose, nano‐titanium oxide, and nanopalladium chloride for the determination of VLD. The study established that VLD was identified at a detection limit of 8.79 × 10^−10^ M, and the method devised was also utilized for pharmaceutical dosage forms and urine samples [9]. Hussien et al. determined VLD utilizing a pencil graphite electrode with a detection range of 6.4 × 10^−9^ M, conducting the method in pharmaceutical dosage forms and human plasma [10]. The study also proposed a mechanism for the oxidation of VLD. While research has yielded a sensitive method for VLD, the fabrication of customized electrodes necessitates a series of pretreatments and preparations. Furthermore, while pencil graphite electrodes offer benefits related to simplicity of application and affordability, they exhibit drawbacks such as low reproducibility and repeatability, as well as mechanical fragility, resulting in numerous obstacles [11, 12]. Consequently, boron‐doped diamond (BDD) electrodes have the potential to be utilized for the determination of VLD given their demonstrated stability, sensitivity, repeatability, and reproducibility.

As technological advancements continue and the demand for sensors escalates, electrochemical sensors are progressively acquiring substantial significance. Electrochemical sensors are highly versatile and can be used in a wide range of applications because of their sensitivity, low detection limits, ease of implementation, affordability, and portability [13]. A diverse array of electrochemical sensors is currently undergoing development for a myriad of applications. BDD electrodes have also established their role among these sensors. BDD is not merely an electrode material; it is also a boron‐doped semiconductor that exhibits semimetallic characteristics [14]. The broad spectrum of applications of these electrodes is attributable to their electrocatalytic characteristics. The factors that are crucial in the preference for constructing electrochemical sensors include their extensive potential range, minimal background, and capacitance current, resulting in enhanced detection limits, as well as good stability and repeatability [15]. Particularly in surfactant‐containing environments, the BDD electrode’s low tendency for surface adsorption and its structure resistant to electrode passivation enable the acquisition of repeatable and reliable voltammetric responses. A thorough analysis of the extant research reveals that BDDE has applications in a variety of pharmacological active components, pesticides, and organic contaminants, as well as in ensuring food safety [16, 17].

Surfactants are known to influence analyte–electrode interactions and electroanalytical performance by modifying analyte adsorption, voltammetric peak characteristics, signal intensity, and the interfacial environment at the electrode surface [16, 18]. Surfactants can act as a major role in electrochemical sensing by modifying the electrode/solution interface and, as a result, the interplay of the target analyte with the electrode surface. Their adsorption at the electrode interface can influence analyte adsorption, electron‐transfer procedure, redox potentials, diffusion behavior, and also ultimately the magnitude and characteristics of the voltammetric reply [19, 20]. Importantly, these results rely on the ionic character of the surfactant; so, cationic, anionic, and nonionic surfactant species may manufacture distinct electrochemical responses for the same analyte. Comparative works employing CTAB, SDS, and nonionic/neutral surfactants at BDD electrodes have indicated this surfactant‐dependent behavior, with SDS‐containing media ensuring enhanced anodic responses for the investigated pharmaceutical compounds [19, 20]. Thus, appropriate selection of both surfactant type and concentration represents an important strategy for controlling analyte–electrode interactions and improving voltammetric signal characteristics in BDD‐based pharmaceutical analysis.

In this study, a new, fast, and highly sensitive electrochemical method based on surfactant‐supported differential pulse voltammetry (DPV) using a BDD electrode has been developed for the quantitative determination of VLD for the first time. The investigation of the effects of surfactants with different ionic properties on the electrochemical behavior of vilazodone provides novel signal enhancement ideas based on the control of electrode–analyte interactions, contributing original insights to the literature. The results also demonstrate that combining the BDD electrode with surfactant‐mediated medium optimization enables microlevel analysis in complex biological matrix environments and pharmaceutical dosage forms. Furthermore, a method has been developed that is easy to prepare and apply, has high sensitivity and high reproducibility compared to methods developed for VLD determination.

## 2 Experimental

### 2.1 Reagents and Chemicals

VLD and its tablet dosage form (ZILADONE) were supplied from NOBEL Holding A.S. (Düzce, Turkey). TWEEN 20, CTAB (≥98%), SDS (≥99%), glacial acetic acid (≥99%), sodium acetate trihydrate (≥99.5%), methanol (≥99.8%), boric acid (≥99.5%), phosphoric acid (≥85%), sodium phosphate dibasic (≥99%), sodium hydroxide (≥97.0%, pellets), sodium chloride (≥99.0%), potassium chloride (99.0%–100.5%), hydrochloric acid (37%), dimethyl sulfoxide (DMSO) (≥99.7%), glucose, ascorbic acid, dopamine hydrochloride (≥98.0%), caffeine (anhydrous, 99%), and commercial human serum samples (AB, male were purchased from Sigma Aldrich (USA).

All solution was prepared using a Millipore Milli‐Q system with analytical‐grade chemicals and double‐distilled water.

### 2.2 Preparation of Solutions

The following were utilized as supporting electrolytes: Britton‐Robinson buffer solution (BRBS) (pH 2.00–12.00), phosphate buffer (pH 6.00–8.00), acetate buffer (pH 3.60–5.65), and sulfuric acid (0.1 M, pH 2.00).

A stock solution of 1 × 10^−3^ M VLD was formulated in DMSO. The necessary volumes of the stock solution were diluted with BRBS (pH 4.0) as a supporting electrolyte while keeping a consistent concentration of 10% DMSO.

The surfactants evaluated include SDS, CTAB, and TWEEN 20, which were produced by dissolving the required quantities of reactants in water.

### 2.3 Instrumentation and Apparatus

The AUTOLAB‐PGSTAT 204 (The Netherlands) and NOVA 2.1.6 software were used for all voltammetric measurements.

Investigations typically use the requirements of three‐electrode configuration, with a platinum wire serving as the counter electrode, an Ag/AgCl reference electrode (3 M NaCl), and a boron‐doped diamond (BDD, Windsor Scientific, Ø: 3 mm, diameter) as the working electrode.

The buffer solutions’ pH levels were adjusted with the use of a SevenCompactTM pH/Ion S220 model (Mettler Toledo, Switzerland) that was equipped with a setup of reference electrodes made of glass.

The BDD working electrode was polished using an alumina slurry on a polishing pad to achieve a reproducible and electrochemically active surface. Following polishing, the electrode was thoroughly rinsed with distilled water to remove residual alumina particles. This procedure provided a consistent and active surface for all subsequent electrochemical measurements. Since the electrochemical response of BDD is highly dependent on its surface condition, mechanical polishing was performed gently and under identical conditions throughout the study to minimize possible surface alterations while providing a clean and reproducible electroactive surface [19, 20].

### 2.4 Recovery Studies With Commercial Serum Samples and Pharmaceutical Dosage Forms

Samples of commercial human serum that had been frozen at −20 °C in a refrigerator were stored at room temperature. 3.6 mL of serum, 5.4 mL of acetonitrile, and 1.0 mL of VLD stock solution (1.0 × 10^−2^ M) were mixed together in a 10.0 mL centrifuge tube and subjected to ultrasound treatment for 15 min. The mixture was centrifuged at 3500 rpm for 15 min, resulting in a supernatant that was meticulously collected. Serum measurements were conducted utilizing the clear supernatant following dilution with an electrolyte solution.

The average weight of 10 tablets, each containing 10 mg of VLD, was recorded before their complete pulverization for analysis. An equivalent of 1 × 10^−3^ M VLD of this powder was precisely weighed and diluted to 100 mL with DMSO in a volumetric flask. The sample was agitated in an ultrasonic bath for 30 min. An adequate volume of the upper clear solution was subsequently added to the tube containing the chosen support electrolyte.

## 3 Results and Discussion

### 3.1 Electrochemical Behavior of VLD on BDD Electrode

#### 3.1.1 Influence of pH

To examine the impact of pH on the VLD oxidation signal at the unmodified BDD, 1.0 × 10^−4^ M VLD solutions were prepared at different pH ratings, and measurements were conducted. In BRBS (pH 2.0–12.0), phosphate buffer solution (6.02–7.02), and acetate buffer solution (3.7–5.7), the electrochemical behavior of VLD was examined. Figure 1 demonstrates the influence of pH on peak potential and peak current. Although a higher current response was observed in pH 3.0 BRBS, the inadequate peak morphology, poor resolution, and low reproducibility limited the analytical reliability of this medium. In contrast, comparably high peak current values were also obtained at a pH of 4.0, together with improved peak shape, higher resolution, and better reproducibility. Therefore, pH 4.0 BRBS was selected as the supporting electrolyte for subsequent analytical studies.

**FIGURE 1:**
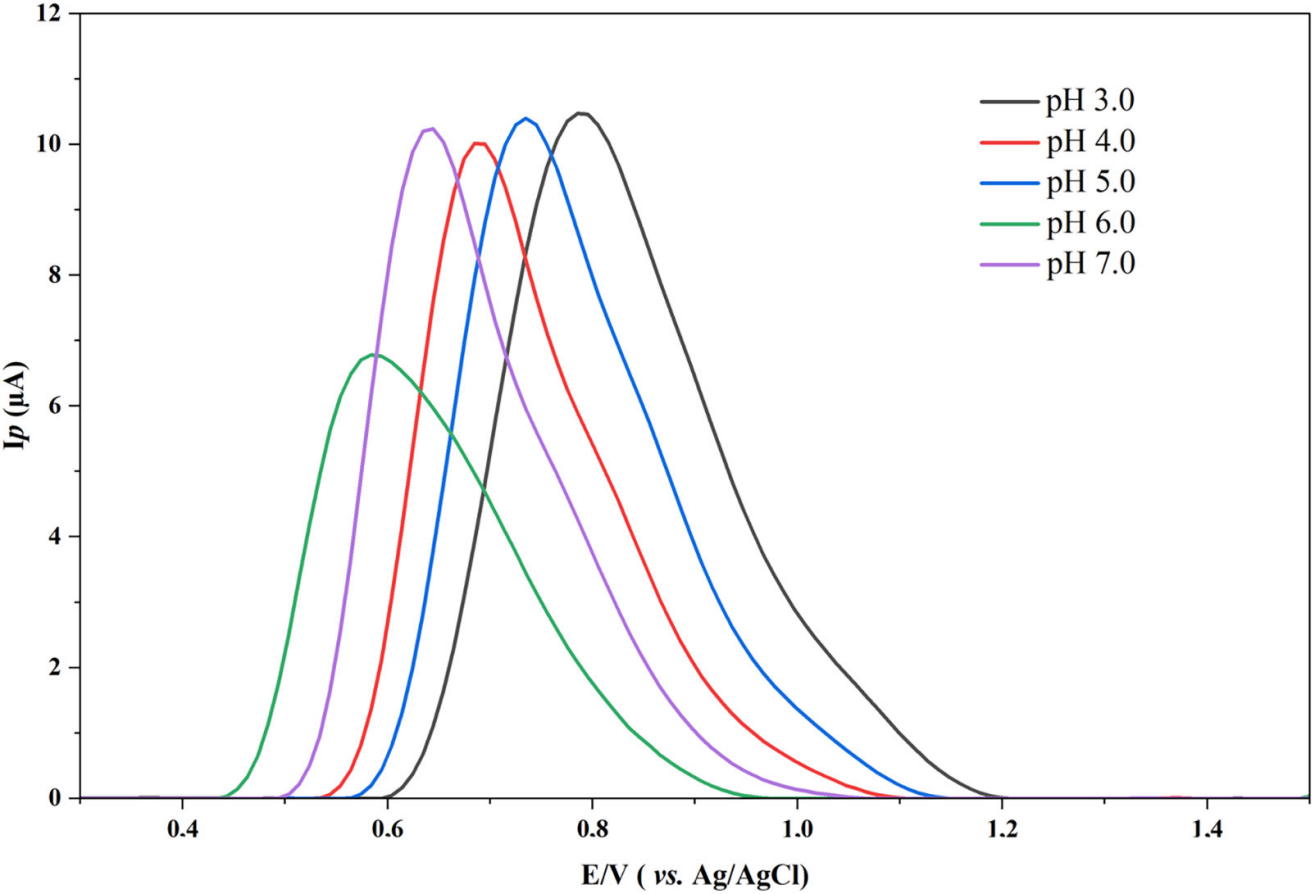
DP voltammograms for 10^−4^ M VLD in BR buffer at pH 3.0–12.0.

The electrochemical behavior of VLD was violently dependent on the pH of the supporting electrolyte. As demonstrated in Figure 2A, the oxidation peak potential shifted progressively toward less positive values with increasing pH up to about pH 7.0. The relation between pH and the peak potential in this area was stated as *E*_p_ (V) = −0.0524 pH + 0.9487. The negative shift in *E*_p_ with increasing pH represents the involvement of protons in the electrooxidation of VLD. Moreover, the slope of −52.4 mV pH^−1^ is very close to the theoretical Nernstian value of −59 mV pH^−1^ at 25 °C for an electrode process involving an equal number of protons and electrons. For that reason, the *E*_p_–pH relationship proposes an approximately 1:1 proton‐to‐electron ratio in the pH‐dependent oxidation process. This indicates that, unlike the mechanism proposed by Hussien et al. for VLD, an equal number of electrons and protons are involved in the oxidation mechanism [10].

**FIGURE 2:**
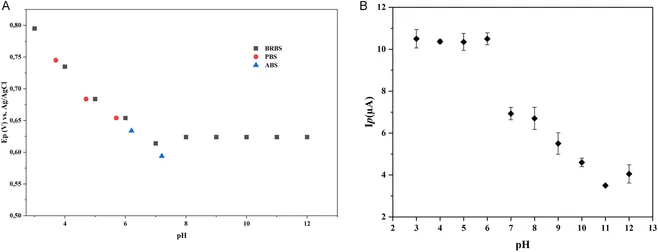
Effect of pH on the 1.0 × 10^−4^ M VLD peak potential (A) and peak current (B) using DPV at the bare BDD electrode.

A clear alteration in the *E*_p_–pH behavior was noticed at approximately pH 7.0, above which the oxidation potential became nearly independent of pH. This break point is coherent with the announced pKa value of VLD of approximately 7.0 [21]. Below this pKa, VLD exists mainly in its protonated form, whereas progressive deprotonation happens as the pH approaches and exceeds the pKa. As a consequence, the informed *E*_p_–pH dependence below pH 7.0 assists a proton‐coupled electron‐transfer procedure, while the substantially reduced pH dependence above this area indicates a diminished contribution of proton transfer to the electrode reaction.

The peak current of VLD was also severely affected by pH (Figure 2B), which can be attributed to alterations in the protonation state of VLD and consequently its interplay with the BDD/electrolyte interface. Although a relatively high current reply was noticed at pH 3.0, the peak morphology, resolution, and reproducibility were less satisfactory. In contrast, pH 4.0 ensured a comparably high current reply together with a well‐defined peak and improved reproducibility. Accordingly, considering both the electrochemical response and analytical performance, pH 4.0 BRB was selected as the optimum supporting electrolyte for subsequent measurements.

#### 3.1.2 The Scan Rate Effect

The influence of scan rate (*v*) on peak current *I*_p_ and *E*_p_ was studied in 1.0 × 10^−4^ M VLD produced in pH 4.0 BRBS using cyclic voltammetry (CV) ranging from 10 to 200 mV s^−1^ (Figure 3).

**FIGURE 3:**
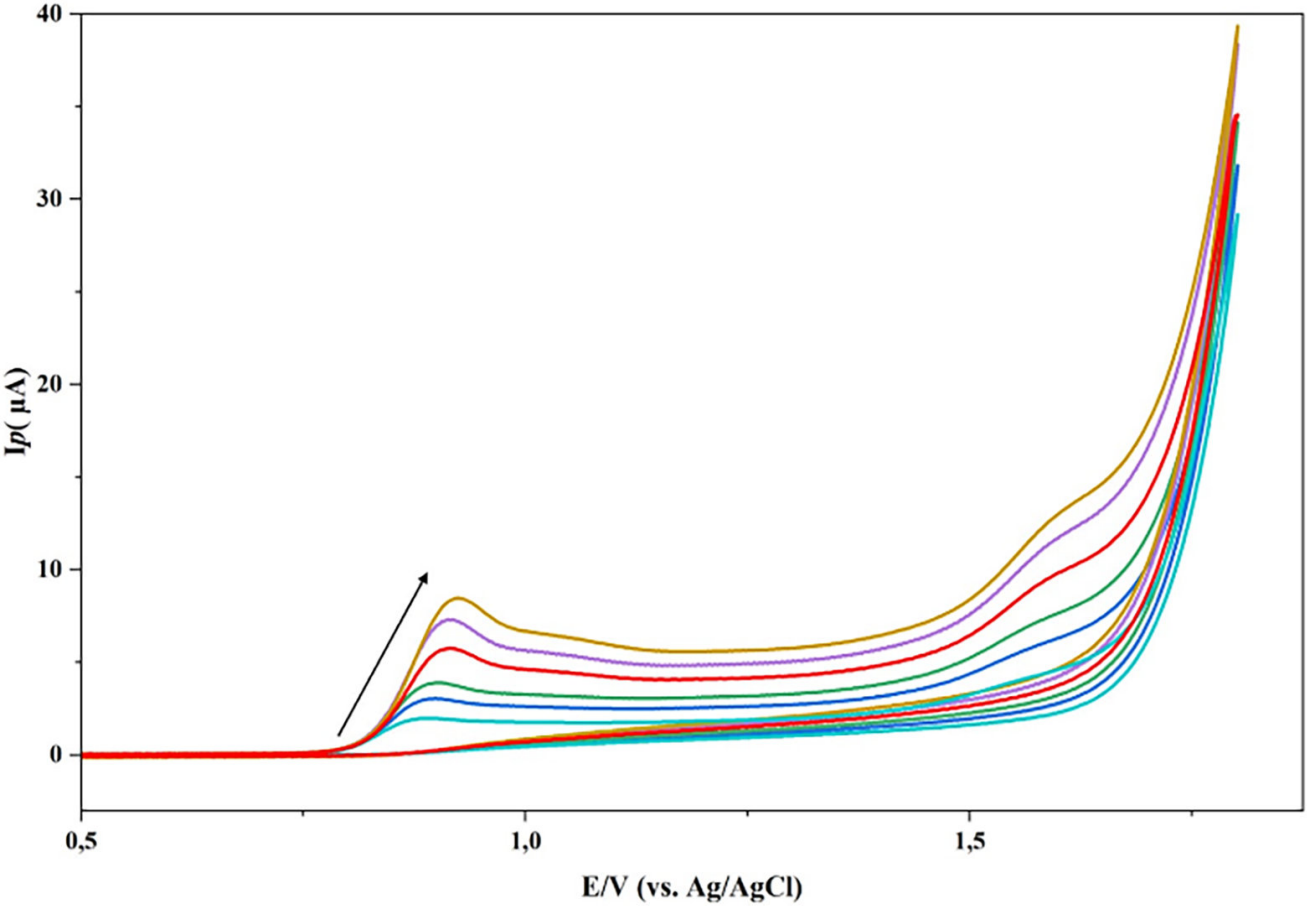
Cyclic voltammogram of 10^−4^ M VLD in pH 4.0 BR buffer at the scan rate range of 10–200 mV s^−1^.

The log *I*_p_ versus log *V* plot was generated based on the *I*_p_ values changing with *v*, and the equation connected to it is as follows [22]

(1) $$\log\;I_{p}\text{ =}\;0\text{.}5761\;\log\;v\;\text{+}\;0\text{.}9456\;\left(r\;\text{=}\;0\text{.}9975;\;n\;\text{=}\;6\right)$$

The knowledge about the control of the transfer process on the electrode surface by diffusion or adsorption can be derived from the slope value of the linear relationship between log *I*_p_ and log *v*. If the slope of the log *I*_p_ versus log* v* plot approaches 0.5, the electron transfer process is diffusion‐controlled; if it approaches 1, it is adsorption‐controlled. The equation above yields a value of 0.57, thereby supporting the diffusion‐controlled mechanism [23]. In addition, it should be recorded that the magnitude of the voltammetric current relies on the electrode area present for interfacial electron transfer. The BDD electrode employed in this search had a circular exposed surface with a diameter of 3 mm, corresponding to a geometrically exposed area of 0.0707 cm^2^ (*A* = *πr* ^2^). Consequently, the voltammetric replies reported herein were obtained utilizing a constant and well‐defined exposed BDD surface area. The importance of the exposed electrode area in interpreting voltammetric replies has also been emphasized in previous electroanalytical studies [24, 25].

The electron‐transfer number (*n*) for the redox reaction was evaluated based on the DP voltammogram results. For VLD at BDDE, the Δ*E*_p_ parameter (*E*_p _ −  *E*_p/2_) was determined as 90.0 mV. This value was then applied to Equation (2) in order to determine the value of *n*.

(2) $$\text{∆}E_{p}=E_{p}–E_{p/2}=(47\text{.}7/\alpha n)\;\mathrm{mV}$$

For electrochemical processes that proceed with fully irreversible characteristics, the electron transfer coefficient (*α*) is typically assumed to be 0.5 [26]. By introducing this value into the related equation, the anodic electron number (*n*_a_) was calculated as 1.59, which can be approximated to 2 for the molecule investigated in this study. Similar irreversible electrochemical behavior has also been reported in previous studies.

In the equation, *α* denotes the charge‐transfer coefficient, whereas *n* signifies the number of electrons participating in the reaction. According to this equation, when *E*_p_ is plotted against log *v*, the slope obtained is used to find that*αn* is 1. The *α* value is typically set at 0.5 for irreversible processes. Consequently, the value of n was determined to be roughly 2. This figure signifies that two electrons per molecule participate in the oxidation process of VLD.

After determining the number of electrons involved in the oxidation process of VLD, the number of protons was also calculated. For this purpose, Equation (3) has been used

(3) $$dE_{p}/d\mathrm{pH}=0\text{.}059P/\alpha n$$

The *dE*_p_/*d*pH denotes the slope of the curve derived from plotting pH against oxidation peak potential, whereas *P* signifies the quantity of protons engaged in the process [27].

Based on this relationship, the number of protons has been found to be approximately 2. Based on this data, it can be said that an equal number of electrons and protons are involved in the oxidation process of VLD. This is also confirmed by the fact that when pH values are plotted against oxidation peak potentials, the slope of the resulting curve is 0.92 V. This value indicates that an equal number of electrons and protons are involved in the reaction.

In this study, using a BDD electrode, it was observed that VLD was oxidized with 2 electrons and 2 protons via the piperazine ring, following a different mechanism than in study conducted by Fekry et al. [9], taking into account studies with model drugs [28]. The proposed oxidation mechanism is discussed in detail in Section 3.7.

#### 3.1.3 Influence of Various Surfactants on Electrochemical Reaction

In trials conducted to determine the effect of surfactants, all measurements were carried out by separately adding 1 × 10^−4^ M CTAB, SDS, and Tween 20 to a 1 × 10^−4^ M VLD solution with a constant analyte concentration (Figure 4). This comparison under equal concentration conditions provided an important basis for assessing the actual contribution of surfactants to the electroanalytical response. The obtained DPV results revealed that the addition of Tween 20 led to a significantly higher current increase in the vilazodone oxidation peak compared to other surfactants. This effect was attributed to the nonionic nature of Tween 20, allowing the analyte to more efficiently reach the BDD electrode surface by avoiding the electrostatic interactions created by CTAB and SDS. The evaluation of the voltammetric behavior in the environment containing Tween 20 showed that the vilazodone oxidation process is diffusion‐controlled. The nonionic structure of Tween 20 created a significant adsorption barrier on the electrode surface, preventing excessive binding of the analyte to the surface, thus allowing mass transfer to occur primarily through diffusion in the solution. Additionally, the microcolloidal structure formed by Tween 20 distributed the analyte molecules homogeneously in the solution, suppressing convective and adsorption‐controlled processes and supporting that the current is governed by a diffusion‐limited mechanism.

**FIGURE 4:**
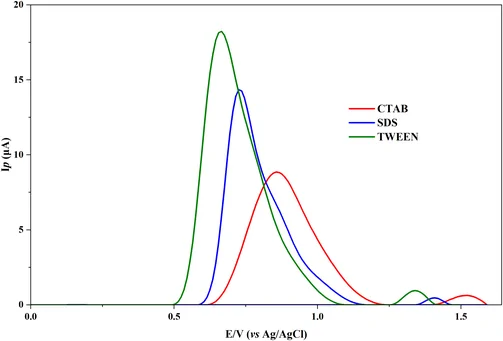
DP voltammograms for the detection of 1 × 10^−4^ M VLD in pH 4.0 BRBS buffer in the presence of various surfactants (10^−4^ M).

#### 3.1.4 The Optimization of Tween 20 concentration

Following the identification of Tween 20 as the surfactant that accelerates the reaction and leads to positive improvements in VLD oxidation, concentration was optimized as another parameter affecting sensor performance. In order to achieve this objective, while the VLD concentration was maintained at a constant level of 10^−4^ M, the Tween 20 concentration was subjected to variation within the range of 2 × 10^−5^ and 8 × 10^−4^ M. As demonstrated in Figure 5, a substantial enhancement in the peak current was observed in the presence of Tween 20. As the Tween concentration increased, the peak current also increased concomitantly until it reached 1 × 10^−4^ M. However, at higher concentrations, a decrease in reproducibility and peak currents was observed. Therefore, the optimum Tween 20 concentration was determined as 1 × 10^−4^ M. Tween 20 has an amphiphilic structure consisting of a hydrophobic laurate chain (fatty acid moiety) and hydrophilic polyoxyethylene chains. The BDD electrode surface may exhibit partially hydrophobic properties depending on the surface modification. This situation can facilitate electron transfer by increasing the accumulation of the analyte on the electrode surface at the critical micelle concentration (0.06 mg/mL) [29], thereby leading to an increase in peak current. Above this concentration (1 × 10^−4^ M), it supports the transport of the analyte to the electrode surface via diffusion through micelle formation. Due to its nonionic structure, Tween 20 contributes only minimally to electrostatic interactions, which are considered to play a secondary role in the adsorption process. Therefore, the primary driving forces of adsorption are attributed to hydrophobic interactions and van der Waals forces.

**FIGURE 5:**
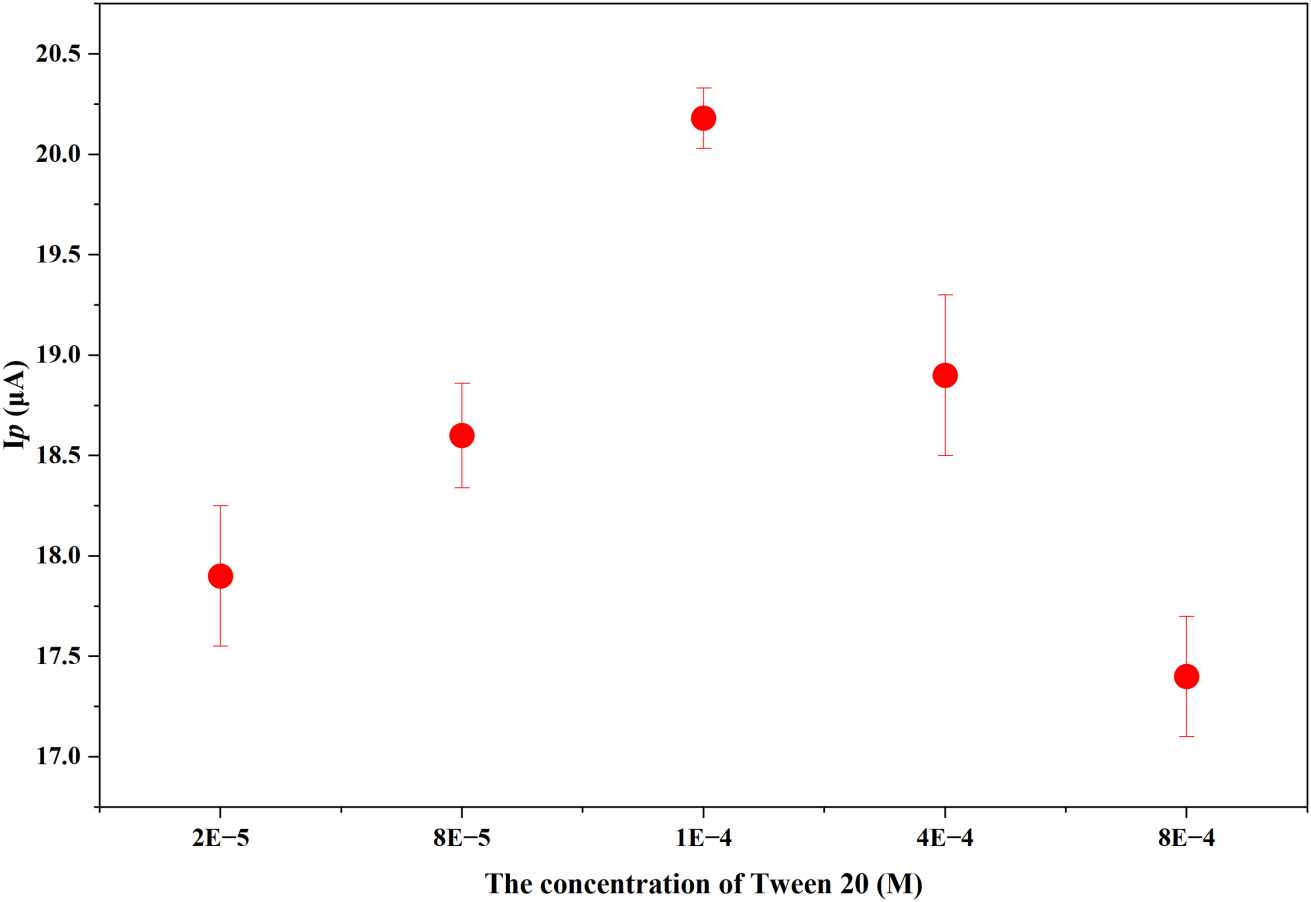
The peak currents obtained for the detection of 1 × 10^−4^ M VLD in the presence of the different concentrations of Tween 20 in pH 4.0 BRB.

### 3.2 Analytical Parameters

DPV was chosen as the primary quantitative method since its pulse‐based measurement reduces the contribution of capacitive background current and ensures a favorable signal‐to‐background ratio, high sensitivity, and good peak resolution, which are particularly benefits for trace‐level determination of VLD. In the present study, CV was essentially utilized to investigate the electrochemical behavior and oxidation mechanism of VLD, whereas DPV was used for quantitative analysis owing to its suitability for detecting low analyte concentrations. Although SWV is also a highly sensitive pulse voltammetric technique, a direct experimental comparison between DPV and SWV was beyond the scope of the present study; therefore, no claim of analytical superiority over SWV is made. Under the optimized conditions, DPV enabled VLD determination down to the sub‐nanomolar level, supporting its suitability for the proposed analytical application.

Using the peak currents around +0.67 V from the experiments shown in the voltammogram in Figure 6, it shows a linear response in the range of 5.0 × 10^−10^–1.0 × 10^−6^ M. The regression equation for the calibration graph is given as *i*_p_ (µA) = 675 948 *C* (nM)  − 0.0154, *R* ^2^ = 0.9884, where *i*_p_ is the peak current, *C* is the VLD concentration, and *r* is the correlation coefficient. Limit of detection (LOD) and quantification (LOQ) have been calculated using the formulas 3 and 10 s/m, respectively, to ascertain the analytical sensitivity of the voltammetric method used in this study. In this calculation, s stands for standard deviation of the peak current (three replicative measurements) of the calibration curve’s lowest level concentration, and m is the slope of the associated calibration equation. LOD and LOQ were found to be 1.52 × 10^−10^ and 4.55 × 10^−10^, respectively.

**FIGURE 6:**
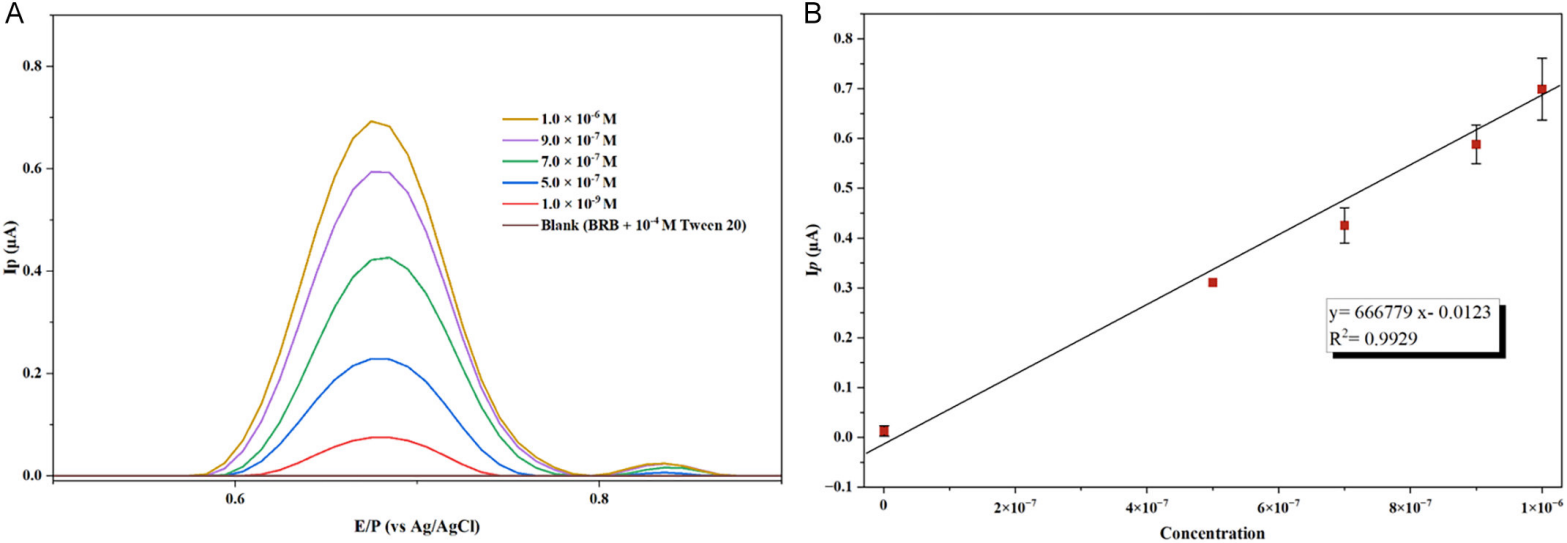
DP voltammograms for standard VLD (A) and graph of various VLD concentrations (B) in 10^−4^ M Tween 20 containing pH 4.0 BRB.

### 3.3 Determination of VLD in Serum Samples and Pharmaceutical Dosage Forms

To demonstrate the applicability of the developed method, real sample analyses were performed on commercial human serum and tablet dosage forms. In the commercial serum sample, the VLD concentration showed linearity in the range of 0.3–1.0 µM, and a calibration curve was plotted within this study range (Figure 7). Analytical parameters of the calibration curve are presented in Table 1. The analytical performance of the developed method was also evaluated using the spike method by adding a 0.7 µM VLD solution to a tablet dosage form solution at a certain concentration. The recovery study was conducted to determine whether VLD interacts with the excipients of the tablet dosage form. The amount of VLD can be recovered close to 100% using the proposed method (Table 1); this indicates that there is no interference effect in pharmaceutical dosage forms. The accuracy and precision of the developed method have been confirmed.

**FIGURE 7:**
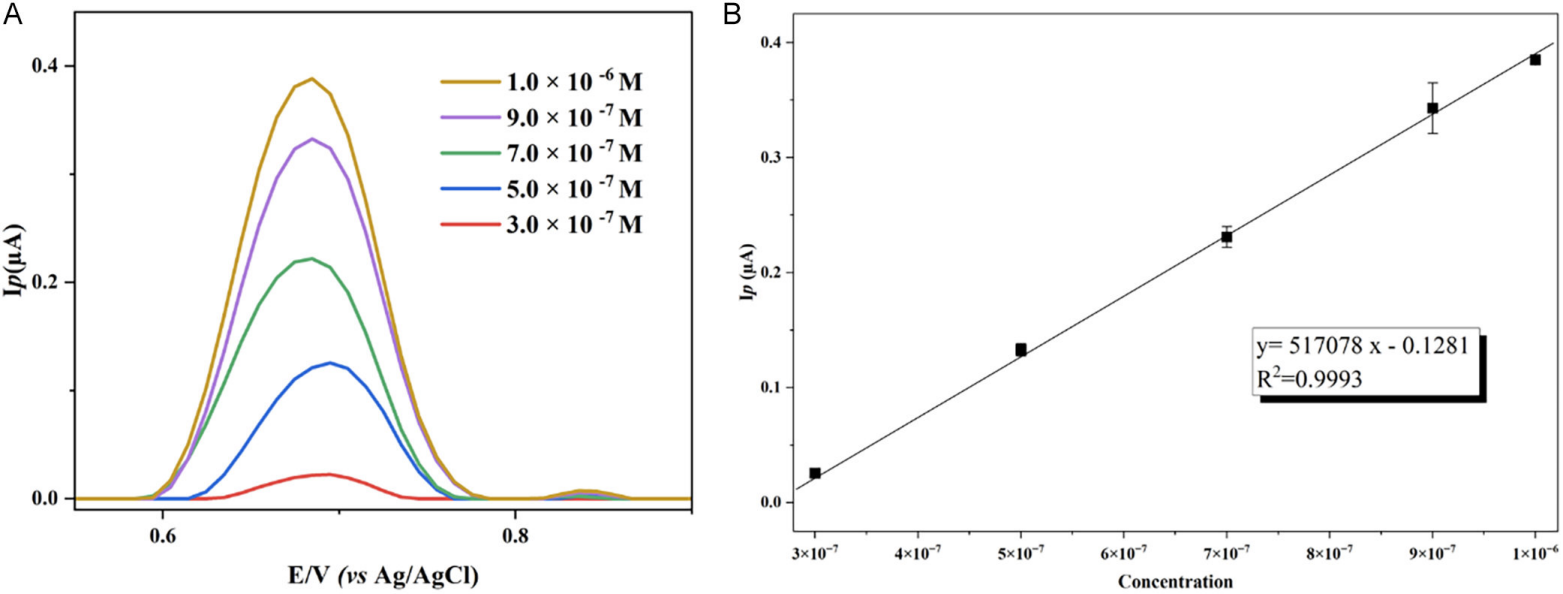
For commercial human serum, DP voltammograms (A) and the plot of various VLD concentrations in pH 4.0 BRBS with 5 × 10^−4^ M Tween 20 (B).

**TABLE 1:** Data obtained from the standard and the calibration chart of the serum.

Parameters	Standard		Serum sample		Tablet dosage form
Linearity range	0.5 nM−1.0 µM		0.3–1.0 µM		—
Correlation coefficient (*r*)	0.9884		0.9993		—
Slope, μA/M	0.6759		0.5171		—
Intercept, μA	0.0154		0.1281		—
LOD	0.152 nM		0.087 µM		—
LOQ	0.455 nM		0.29 µM		—
Added concentration, µM	—		0.7		0.7
Found concentration, µM	—		0.712		0.695
Average recovered %	—		101.79		99.40
RSD% of recovery^a^	—		2.42		1.96
Intraday of peak current repeatability (RSD%)^a^	1.82		—		—
Interday of peak current repeatability (RSD%) a	1.79		—		—

a Each value is the mean of three different measurements.

### 3.4 Interference Study

Interference studies were conducted to evaluate the selectivity of the developed method (Figure 8). It was found that even a 10‐fold higher concentration of inorganic K^+^, Cl^−^, and Na^+^ ions, which are potential interfering compounds frequently encountered in biological matrices, did not affect the VLD current responses. Likewise, common urine components such as ascorbic acid and dopamine, as well as major interfering substances in drug analyses such as caffeine and glucose, were found to have no interfering effect on VLD determination at the same and 10‐fold higher concentration levels. The maximum interferent concentration affecting the electrochemical response of 0.7 µM VLD by less than 5% was determined to be 10‐fold.

**FIGURE 8:**
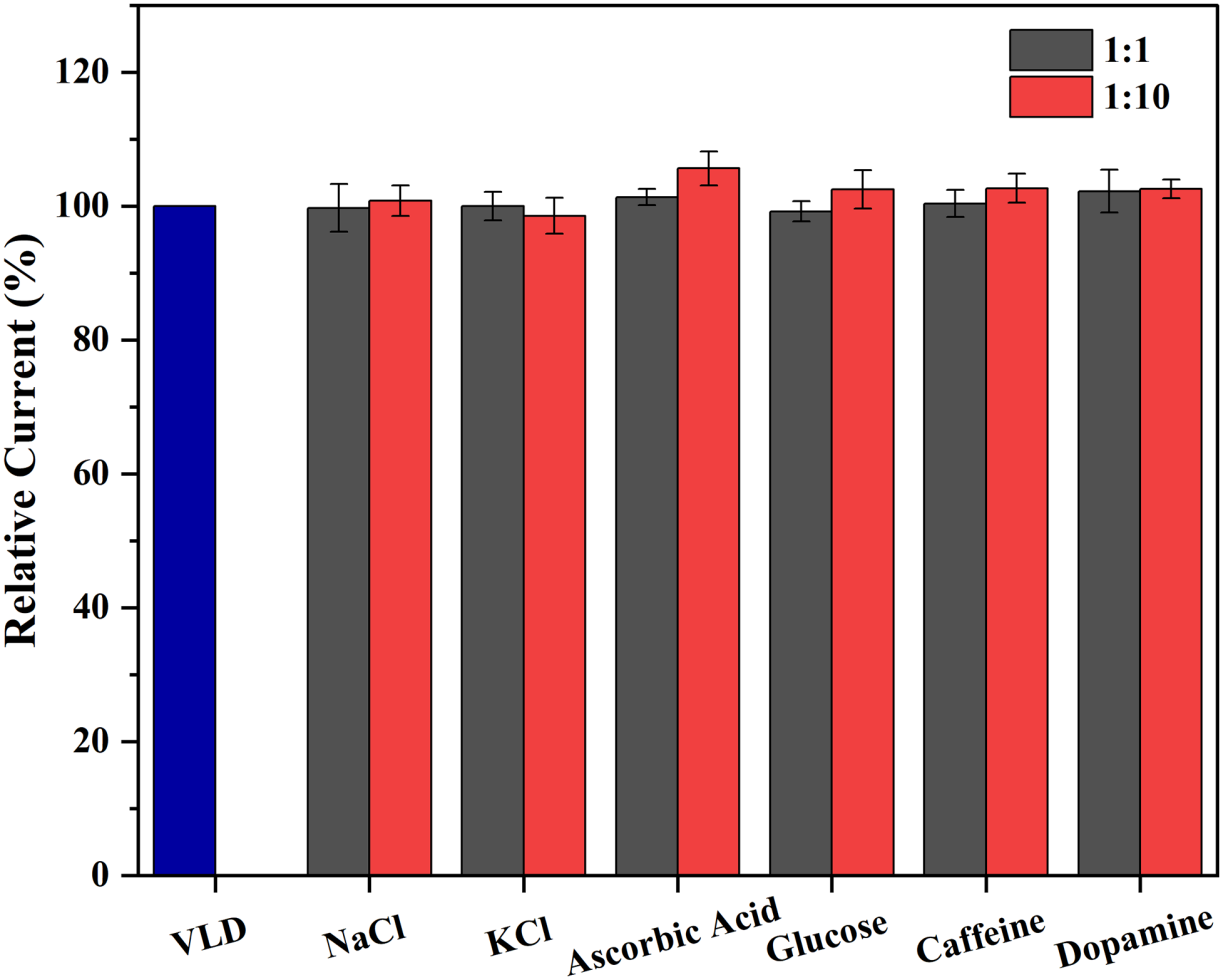
Recovery of 0.7 µM VLD with 1:1 and 1:10 interference compounds.

### 3.5 DFT Calculations

Ground‐state geometric optimization calculations were governed by utilizing Density Functional Theory (DFT). Computational calculations and visualizations regarding the VLD were fulfilled using Gaussian 09 Rev. C.01 and GaussView 5.0.9, respectively. The hybrid B3LYP functional with the 3‐21G basis set was utilized to optimize molecular geometry. These calculations were employed to VLD in the ground state at the gas phase.

### 3.6 DFT Results

In molecular orbital known as MO theory, the highest occupied molecular orbital (HOMO) typifies the uppermost energy level that contains electrons in the ground state and is therefore closely associated with the electron‐donating ability of a molecule. Zones where the HOMO is localized are more prone to participate in oxidation processes, nucleophilic interplays, and electron‐transfer incidents. Consequently, the spatial distribution of the HOMO provisions direct insight into the most reactive electron‐rich sites of a target molecule. Otherwise, the lowest unoccupied molecular orbital (LUMO) corresponds to the lowest available energy level that can accept electrons and is related to the electron‐accepting ability of the molecular framework. Localization of the LUMO recognizes molecular zones that are more susceptible to reduction processes or electrophilic interplays [29, 30]. Together, the HOMO and LUMO explain the frontier orbitals that govern chemical reactivity and intermolecular interplays. Optimized structure and HOMO–LUMO orbital distributions of VLD calculated at the B3LYP/3‐21 G level in the gas phase were given in the Figures 9 and 10, respectively.

**FIGURE 9:**
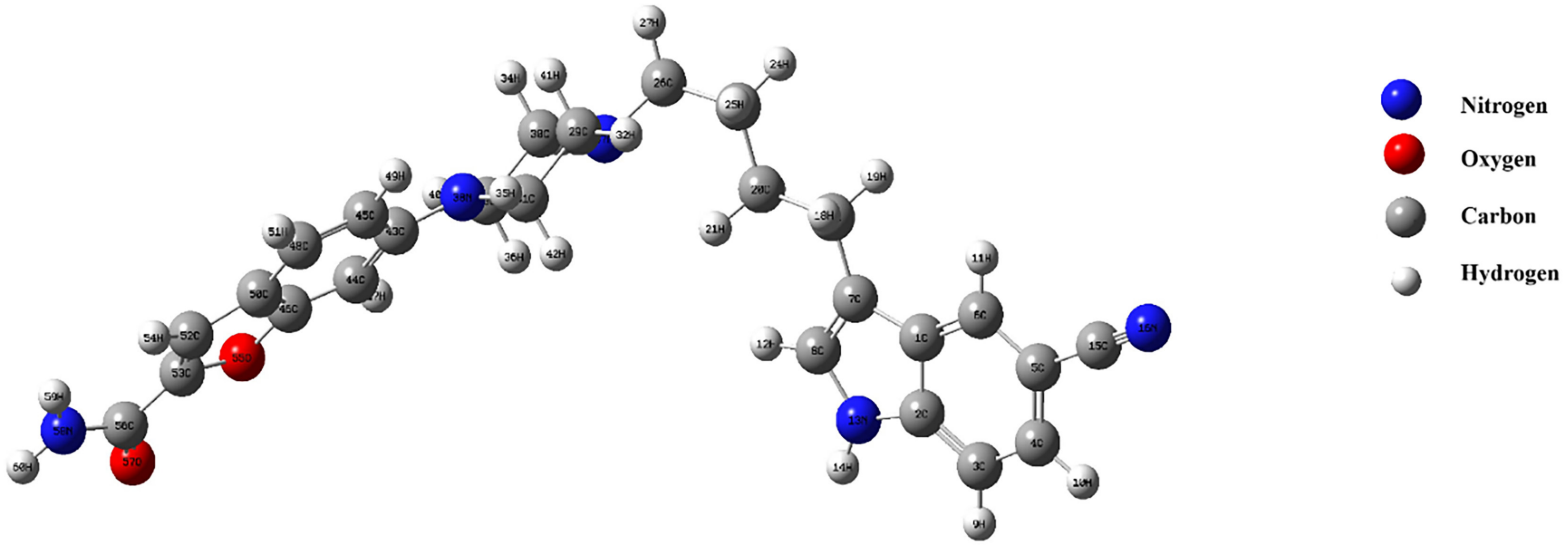
Optimized structure of VLD calculated at the B3LYP/3‐21G level in the gas phase.

**FIGURE 10:**
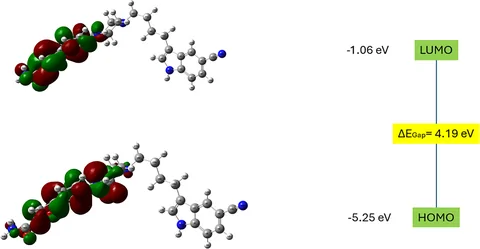
HOMO–LUMO orbital distributions of VLD calculated at the B3LYP/3‐21G level in the gas phase.

Using the computed frontier orbital energies (*E*_HOMO_ = −5.25 eV and *E*_LUMO_ = −1.06 eV), the resulting HOMO–LUMO energy separation of ≈4.19 eV (≈0.154 a.u.) reflects a moderate energy gap between the occupied and unoccupied electronic states of VLD. Such an energy gap is characteristic of molecules with intermediate electronic softness, indicating that the redistribution of electron density does not require excessive energetic input [31]. As shown in Figure 10, the HOMO distribution was mainly located over the nitrogen‐containing conjugated area and partially extended onto the piperazine moiety, proposing that this electron‐rich site may participate in the initial oxidation process. This interpretation is consistent with the electrochemical oxidation pathway previously suggested by Hussein et al., in which the piperazine nitrogen undergoes one‐electron oxidation to form a nitrogen‐centered radical cation, followed by a further electron‐transfer/proton‐loss step. Therefore, the DFT‐derived frontier orbital distribution supports, without being conclusive, the involvement of the piperazine nitrogen in the anodic oxidation of the molecule [10].

### 3.7 Proposed Electrochemical Oxidation Mechanism of VLD

The electrochemical oxidation mechanism of VLD at the BDD electrode was appraised by correlating the experimental voltammetric outcomes with the electronic–structure knowledge acquired from DFT calculations. The CV behavior demonstrated an anodic oxidation reply without a corresponding cathodic peak on the reverse scan, showing the irreversible nature of the electrode process. Furthermore, the oxidation peak potential shifted toward less positive values with increasing pH up to approximately pH 7.0. The slope of the Ep–pH relationship (−52.4 mV pH^−1^) was very close to the theoretical Nernstian value of −59 mV pH^−1^ supposed for an electrode process involving an approximately equal number of protons and electrons. Consequently, the pH dependence ensures experimental proof that proton transfer is coupled to electron transfer during VLD oxidation.

Additionally, mechanistic data was acquired from the electrochemical determination of the number of transferred electrons. The Δ*E*_p_ (*E*_p_−*E*_p/2_) value of 90.0 mV, together with a transfer coefficient (*α*) of 0.5 for the irreversible process, yielded an electron‐transfer number of 1.59, which was approximated to two electrons. Combined with the approximately 1:1 proton–to–electron relationship derived from the *E*_p_–pH dependency, these evidence promotes an overall two‐electron/two‐proton (2e^−^/2H^+^) oxidation process. Besides, the slope of 0.5761 obtained from the log *I*_p_ versus log *v* relationship was close to the theoretical value of 0.5 for a diffusion‐controlled process, indicating that transport of VLD from the bulk solution to the BDD surface predominantly governs the overall electrode response.

The DFT computations ensure molecular‐level understanding into the experimentally suggested oxidation pathway. The calculated frontier molecular orbital energies were *E*_HOMO_ = −5.25 eV and *E*_LUMO_ = −1.06 eV, giving a HOMO–LUMO energy gap of about 4.19 eV. More importantly for the anodic procedure, the HOMO electron density was mainly distributed over the nitrogen‐containing conjugated region and partially extended onto the piperazine moiety. Since anodic oxidation necessitates electron removal from an occupied molecular orbital, the HOMO distribution determines this nitrogen‐rich region as a reasonable electron‐donating site. In contrast, the LUMO defines the lowest‐energy orbital available for electron acceptance and, whence has less direct mechanistic relevance to the experimentally observed anodic oxidation. In spite of that, reflection of both frontier orbitals ensures an overall description of the electronic distribution and reactivity of VLD [29, 30, 31].

On the basis of these complementary findings, the piperazine‐containing region is proposed as the principal electroactive site of VLD. The oxidation may be initiated by one‐electron removal from the electron‐rich piperazine nitrogen to generate a nitrogen‐centered radical cation. Subsequent deprotonation, followed by a second electron‐transfer/deprotonation step, can account for the experimentally determined overall 2e^−^/2H^+^ process. The susceptibility of piperazine nitrogen centers to electrochemical oxidation has been demonstrated in previous electrochemical investigations of piperazine derivatives [27], and a piperazine‐centered oxidation pathway has also been proposed specifically for VLD [10]. Therefore, the localization of the HOMO over the nitrogen‐rich/piperazine‐containing region provides an independent theoretical rationale for the experimentally derived oxidation pathway rather than merely reproducing the previously reported mechanism.

Overall, the proposed mechanism is supported by mutually consistent experimental and theoretical observations: (i) the irreversible anodic voltammetric behavior, (ii) the pH‐dependent potential shift indicating proton‐coupled electron transfer, (iii) the experimentally estimated two‐electron/two‐proton stoichiometry, and (iv) the HOMO localization over the nitrogen‐rich region associated with the proposed oxidation site. Thus, DFT calculations complement the voltammetric findings by identifying the molecular region from which electron removal is electronically favorable and provide a mechanistic link between the calculated electronic structure of VLD and its experimentally observed oxidation behavior at the BDD electrode. Nevertheless, since the oxidation product was not structurally characterized in the present study, the pathway shown should be considered a proposed electrochemical mechanism rather than definitive identification of the oxidation product.

## 4 Conclusion

A rapid, highly sensitive, and reliable electrochemical method was developed for the quantitative determination of vilazodone, a novel antidepressant widely prescribed for the treatment of BDDE. The proposed method employs DPV using a BDD electrode, which offers a wide potential window, low background current, and high resistance to surface fouling, making it particularly suitable for trace‐level pharmaceutical and biological analysis.

The effects of various surfactants, including cationic CTAB, anionic SDS, and nonionic Tween 20, were systematically investigated to enhance the electrochemical response. Additionally, several buffer systems were evaluated to optimize the electrochemical environment. Optimization studies demonstrated that the combination of BRBS with Tween 20 produced the most pronounced and reproducible oxidation peak at approximately +0.67 V, resulting in a significant increase in peak current and an improved signal‐to‐noise ratio. The enhancement is attributed to favorable adsorption and preconcentration effects of the surfactant on the BDD electrode surface, which facilitate electron transfer and improve sensitivity. The oxidation mechanism of VLD was investigated using the developed method, and a two‐electron and two‐proton mechanism for the reaction was proposed. Furthermore, VLD was identified at a detection limit of 0.152 nM, and the LOQ value was calculated as 0.455 nM. The successful application of the method to pharmaceutical dosage forms and commercial human serum samples demonstrates its potential as an alternative method for rapid and precise clinical applications.

## Funding

This study was supported by Ankara University Scientific Research Projects Coordination Unit (TSA‐2025‐4087).

## Conflicts of Interest

The authors declare no conflicts of interest.

## Data Availability

The data that support the findings of this study are available from the corresponding author upon reasonable request.
